# Signal functions for measuring the ability of health facilities to provide abortion services: an illustrative analysis using a health facility census in Zambia

**DOI:** 10.1186/s12884-016-0872-5

**Published:** 2016-05-14

**Authors:** Oona M. R. Campbell, Estela M. L. Aquino, Bellington Vwalika, Sabine Gabrysch

**Affiliations:** Faculty of Epidemiology and Population Health, London School of Hygiene and Tropical Medicine, Keppel Street, London, WC1E 7HT UK; Universidade Federal da Bahia, Instituto de Saúde Coletiva, MUSA-Programa Integrado em Gênero e Saúde, Salvador, Bahia Brazil; Department of Obstetrics and Gynaecology, University of Zambia, Lusaka, Zambia; Institute of Public Health, Ruprecht-Karls-Universität, Heidelberg, Germany

**Keywords:** Abortion, Signal functions, Health facility assessments, Health services, Geographic access

## Abstract

**Background:**

Annually, around 44 million abortions are induced worldwide. Safe termination of pregnancy (TOP) services can reduce maternal mortality, but induced abortion is illegal or severely restricted in many countries. All abortions, particularly unsafe induced abortions, may require post-abortion care (PAC) services to treat complications and prevent future unwanted pregnancy. We used a signal-function approach to look at abortion care services and illustrated its utility with secondary data from Zambia.

**Methods:**

We refined signal functions for basic and comprehensive TOP and PAC services, including family planning (FP), and assessed functions currently being collected via multi-country facility surveys. We then used the 2005 Zambian Health Facility Census to estimate the proportion of 1369 health facilities that could provide TOP and PAC services under three scenarios. We linked facility and population data, and calculated the proportion of the Zambian population within reach of such services.

**Results:**

Relevant signal functions are already collected in five facility assessment tools. In Zambia, 30 % of facilities could potentially offer basic TOP services, 3.7 % comprehensive TOP services, 2.6 % basic PAC services, and 0.3 % comprehensive PAC services (four facilities). Capability was highest in hospitals, except for FP functions. Nearly two-thirds of Zambians lived within 15 km of a facility theoretically capable of providing basic TOP, and one-third within 15 km of comprehensive TOP services. However, requiring three doctors for non-emergency TOP, as per Zambian law, reduced potential access to TOP services to 30 % of the population. One-quarter lived within 15 km of basic PAC and 13 % of comprehensive PAC services. In a scenario not requiring FP functions, one-half and one-third of the population were within reach of basic and comprehensive PAC respectively. There were huge urban-rural disparities in access to abortion care services. Comprehensive PAC services were virtually unavailable to the rural population.

**Conclusions:**

Secondary data from facility assessments can highlight gaps in abortion service provision and coverage, but it is necessary to consider TOP and PAC separately. This approach, especially when combined with population data using geographic coordinates, can also be used to model the impact of various policy scenarios on access, such as requiring three medical doctors for non-emergency TOP. Data collection instruments could be improved with minor modifications and used for multi-country comparisons.

## Background

Abortions are induced in every country in the world, with an estimated 43.8 million taking place worldwide in 2008 [1]. Globally, just over half of induced abortions are provided via safe termination of pregnancy (TOP) services, while in Africa only 3 % of induced abortions are safe [1]. The TOP methods recommended by the World Health Organization in first trimester pregnancy (up to 12–14 weeks) are vacuum aspiration or medication abortion (mifepristone followed by a single dose of misoprostol (up to 9 weeks) or multiple doses after 9 weeks)), while after 12–14 weeks, the method of choice is mifepristone followed by multiple doses of misoprostol, or dilation and evacuation (D&E). Where mifepristone is unavailable, repeated doses of misoprostol alone can be used. Dilation and curettage (D&C) is considered obsolete but is common in many settings for first trimester abortions [2, 3]. All abortions, but particularly unsafe induced abortions, may require post-abortion care (PAC) services to treat complications; vacuum aspiration and misoprostol are recommended to remove retained products of conception, and uterotonics or parenteral antibiotics, or even blood transfusions or surgery may also be needed in the most extreme cases. Also, family planning (FP) provision should be integrated with both TOP and PAC, to ensure that subsequent unwanted pregnancies do not ensue, since most women inducing abortion, whether safely or unsafely, do not want another pregnancy in the immediate future [4]. All countries need to plan for PAC services, and where legal, for TOP services as well. However, while the legal situation with respect to abortion in low- and middle-income countries is well documented [5], relatively little is known about the levels of provision of abortion services in health systems or health facilities on a national scale. Existing studies of provision have used a variety of approaches, including, for example, expert opinion in a multi-country study on the extent (out of 100) to which countries provide PAC or TOP services [6], telephone assessments in a study in South Africa [7], a count of facilities that “should” be able to provide abortion care services in a study of India, Nicaragua, Ethiopia, Kenya and South Africa [8], and bespoke tools to assess abortion services in nationally- or sub-nationally representative samples of facilities in Bangladesh, Timor-Leste, Cambodia, and Ethiopia [6, 9–15].

In 1986, the World Health Organization (WHO) described key obstetric functions hospitals ought to be able to provide [16]; these included the ability to care for abortion complications and provide FP support. In 1997, the United Nations (UN) provided guidance on measuring eight Emergency Obstetric Care (EmOC) signal functions [17]. EmOC is subdivided into six medical functions (removal of retained products, parenteral antibiotics, parenteral oxytocic drugs, parenteral anticonvulsants, manual removal of placenta, and assisted vaginal delivery) that form basic EmOC, and a further two functions indicating surgical capability (caesarean section and blood transfusion) that, together with the six medical functions, form comprehensive EmOC. These basic and comprehensive levels roughly correspond to the health centre level (with midwives) and the first-referral hospital level (with medical doctors) respectively. The UN Guidelines also propose benchmarks for numbers of EmOC facilities needed per 500,000 population. The concept of using a subset of functions to signal the ability of health services to address obstetric complications is now widely accepted.

In 2006, Healy and colleagues put forward signal functions for basic and comprehensive safe abortion care (SAC) [8]. Basic SAC includes the ability, during regular outpatient hours, to perform induced abortion for uterine sizes ≤12 weeks for all legal indications, and to provide post-abortion contraception. Furthermore, on a 24-hours per day, 7-days per week (24/7) basis, basic SAC services should be able to remove retained products for uterine sizes of ≤12 weeks, administer essential antibiotics, intravenous replacement fluids and oxytocics, and provide post-abortion contraception [8]. Comprehensive SAC includes an ability to perform all basic SAC functions and to induce abortion for uterine sizes of >12 weeks, for all legal indications [8]. Furthermore, comprehensive SAC services should, on a 24/7 basis, be able to remove retained products for uterine sizes >12 weeks and perform blood transfusion and laparotomy [8]. Healy and colleagues also propose a benchmark of “five facilities per 500,000 population, at least one of which is comprehensive” [8], identical to the UN Guidelines’ minimum EmOC requirement. They present data on this benchmark for India, Nicaragua, Ethiopia, Kenya and South Africa, reporting 100 % of requirement met in India, Nicaragua, Ethiopia, Kenya, and 75 % in South Africa. This approach was subsequently applied by Belton and colleagues in Timor-Leste [13], by Huda and colleagues in Bangladesh [14] and by Otsea and colleagues [9] and Abdella and colleagues in Ethiopia [15]. The last three studies report coverage levels in relation to benchmarks and detail the proportions of hospitals and primary-level facilities that can provide the individual signal functions. Moreover, because they assessed actual capability (rather than designated capability), coverage in Ethiopia was not 100 % of the benchmark as reported by Healy and colleagues, but rather an initial 39 %, increasing to 86 % over time, while coverage in Bangladesh was 0 % increasing to 4 %.

Fortney reports that WHO recommended such extensions to the signal function approach [18]. Indeed other researchers have proposed and used signal functions for child health care [19], emergency neonatal care [20–22], antenatal care [23], and routine delivery care for mothers and newborns [21, 22, 24], and argue that signal functions are useful in general for:

- assessing health system outputs and capability to provide preventive and curative services,
- monitoring trends over time to assess improvements or deterioration in services,
- assessing geographic access to services; and
- studying regional and urban/rural disparities [17, 20, 25, 26].

In this paper, we present a modified signal function approach for abortion care that allows use of existing multi-country facility survey instruments and separates services for terminating pregnancy (TOP) from those for managing complications (PAC) at basic and comprehensive service levels. We then applied this approach to secondary data from Zambia, a country where sufficient detail on health facilities is available at the national level, to illustrate its potential use. Specifically, we 1) characterized Zambian health facilities’ potential ability to provide TOP and PAC services at basic and comprehensive levels under three different assumptions, and 2) linked facility with population census data to calculate the proportion of the population that could access TOP and PAC services at various levels within a certain distance, nationally and by urban/rural place of residence.

## Methods

### Signal function approach

We defined two levels of TOP and PAC services: basic and comprehensive, corresponding typically to hospitals and health centre services. Table 1 presents our criteria compared to the signal functions proposed for SAC by Healy et al. [8].

**Table 1:** Signal function classification system: criteria for termination of pregnancy (TOP) and post-abortion care (PAC) in comparison to previously suggested SAC criteria

	TOP capability		PAC capability		SAC capability^a^	
	Basic	Comp	Basic	Comp	Basic (≤12 weeks)	Comp (>12 weeks)
Vacuum aspiration	X	X			X	X
Medication abortion	X	X			X	X
Dilation & Curettage (D&C)						X
Dilation & Evacuation (D&E)		X				
Removal of retained products			X	X	X	X
Parenteral antibiotics			X	X	X	X
Uterotonics			X	X	X	X
Intravenous fluids			X	X	X	X
Blood transfusion				X		X
Surgical/laparotomy capability				X		X
Contraceptives (condoms + pills + injectables)	X	X	X	X		
Long-acting reversible contraceptives (LARCs): implants or IUDs		X		X		
Family planning at least once per week	X	X				
Family planning 7 days a week			X	X	X	X
Facility open 24 h per day, 7 days a week (24/7)			X	X	X	X
1+ health professionals on duty	X	X	X		^a^	^a^
3+ health professionals registered (needed for 24/7 PAC service)			X		^a^	^a^
1+ medical doctors on duty				X		^a^
3+ medical doctors registered (needed for 24/7 PAC service)				X		^a^
Communication means or referral capacity (for facilities without comprehensive PAC)	X	X	X			

*comp* comprehensive, *iv* intravenous, *IUD* intrauterine device

^a^Criteria for safe abortion care (SAC) as defined by Healy and colleagues [8] are shown for comparison. In their classification system, staffing is implied by having service provision 24/7 but not stated explicitly

Table 2 shows whether pertinent data were collected in five health facility data collection efforts identified in our previous work [24] and by others [27, 28]. Three other potential data collection tools (Rapid Health Facility Assessment, Quick Investigation of Quality, and Service Availability Mapping) were also examined, but discarded because they did not contain enough relevant aspects of TOP, PAC or EmOC, or of FP [29–31]. Table 2 also shows how our criteria were operationalized using data from the 2005 Zambia Health Facility Census (HFC). The HFC, a national-level assessment of health system assets, was developed by the Japan International Cooperation Agency [32] and covered 1421 facilities in Zambia, comprising all public and semi-public facilities (e.g., mission or non-governmental organizations), and some larger private-for-profit facilities. Data collection was done face-to-face by trained members from Zambian District Health Management Teams, supervised by personnel from the Provincial Health Office and by a National Steering Committee. It comprised questionnaires on infrastructure, utilities, equipment, service delivery and human resources, taking one or more days per facility to complete [33, 34].

**Table 2:** Availability of items in selected health-facility assessment instruments, reviewed April 2015, and operationalization in Zambia

	AMDD	SPA	SARA	FASQ	HFC	Operationalization in Zambia HFC
Medication abortion (mifepristone & misoprostol, or misoprostol alone)	*(yes)* ^*1,6*^	*(yes)* ^*6*^	(yes)^a^	no	no	Not asked; measured via ability to remove retained products of conception
Vacuum aspiration	*(yes)* ^*1,b*^	*(yes)* ^*3*^	*(yes)* ^*1,3*^	*(yes)* ^*1*^	*(yes)* ^*1*^	Measured only in facilities providing delivery care; for facilities without delivery services, question on PAC provision used instead
Dilation & Curettage (D&C) – no longer recommended but may still be used as a substitute for vacuum aspiration or misoprostol	*(yes)* ^*1,9*^	*(yes)* ^*3*^	*(yes)* ^*3,4*^	*(yes)* ^*1*^	no	Not asked; measured via ability to remove retained products of conception
Intravenous fluids	*(yes)* ^*5*^	*(yes)* ^*5,6*^	yes	no	*(no)* ^*c*^	Not measured
Removal of retained products	*(yes)* ^*1*^	*(yes)* ^*2*^	*(yes)* ^*1*^	*(yes)* ^*1*^	*(yes)* ^*1*^	Measured only in facilities providing delivery care; for facilities without delivery services, question on PAC provision used instead
Parenteral antibiotics	*(yes)* ^*1*^	*(yes)* ^*1*^	*(yes)* ^*1*^	yes	*(yes)* ^*1*^	
Uterotonics	*(yes)* ^*1*^	*(yes)* ^*1*^	*(yes)* ^*1*^	*(yes)* ^*5*^	*(yes)* ^*1*^	″
Blood transfusion	*(yes)* ^*1*^	yes	yes	yes	*(yes)* ^*1*^	″
Surgical/laparotomy capability	*(yes)* ^*1,7*^	*(yes)* ^*7*^	yes	*(yes)* ^*1,7*^	*(yes)* ^*1,7*^	Measured via caesarean section capability in facilities providing delivery care
Contraceptives (condom + pill + injectable)	*(yes)* ^*1,8*^	yes	yes	yes	yes	Measured
Long-acting reversible contraceptives (LARCs): implant or IUD	*(yes)* ^*1,8*^	yes	yes	yes	yes	Measured
Sterilization	*(yes)* ^*1*^	yes	yes	yes	yes	Measured
Family planning at least once per week	no	yes	no	yes	yes	Measured
Family planning 7 days a week	no	yes	no	yes	yes	Measured
Service provision 24 h per day, 7 days a week (24/7)	*(yes)* ^*1*^	yes	*(yes)* ^*d*^	yes	*(yes)* ^*1*^	Measured only in facilities providing delivery care via question on 24/7 availability of a health professional with midwifery skill. For facilities without delivery services, question on PAC provision used instead
1+ health professionals on duty	*(yes)* ^*1*^	*(yes)* ^*1*^	no	*(yes)* ^*1*^	yes	Measured
3+ health professionals registered (needed for 24/7 PAC service)	yes	yes	yes	yes	yes	Measured
1+ medical doctors on duty	*(yes)* ^*1*^	no	no	*(yes)* ^*1*^	yes	Measured
3+ medical doctors registered (needed for 24/7 PAC service)	yes	yes	yes	yes	yes	Measured
Communication means	yes	yes	yes	yes	yes	Measured
Referral capability	yes	yes	yes	*(yes)* ^*6*^	yes	Measured

Bracketed responses, e.g. (yes), signify there are caveats to the response, as indicated in the footnotes

Zambia measured capability to provide different services as described in the text, mainly by interviewing key informants in facilities, checking inventories, and reviewing records

1 only asked if facility does: deliveries (AMDD or HFC); deliveries or newborn care (SPA); maternal health services (FASC); normal delivery or BEmOC or CEmOC or newborn care services (SARA)

2 only asked if facility does normal delivery, asks if removal of retained products performed after delivery

3 only asked if facility does delivery, asks availability of vacuum aspirator equipment or D&C kit

4 only asked if facility is hospital that offers surgical services (including minor surgery such as suturing, circumcision, wound debridement, etc.) or caesarean section

5 only asked if can treat haemorrhage, not specifically give uterotonics; availability of uterotonic stocks assessed

6 only asked if uses misoprostol to remove retained products

7 caesarean (FASC, HFC); caesarean & minor procedures (SPA); obstetric surgery e.g. caesarean (also asks about operating theatre even if no deliveries) (AMDD)

8 asked if provide temporary FP methods (pills, condoms, injectables, implants, & IUDs) all merged in one response

9 asked if provides D&E to remove retained products

^a^asked for availability of misoprostol tablets, and of emergency contraceptive methods (e.g. levonorgestrel, ulipristal acetate, mifepristone) (merged together); if has FP services, asks if it provided emergency contraceptive services (e.g. levonorgestrel, ulipristal acetate, mifepristone)

^b^only asked if uses vacuum aspiration to remove retained products

^c^only asked about case management for severe pneumonia and severe dehydration for children

^d^asked hours not days open unless facility does caesarean (when 24/7 opening is assessed)

*AMDD* averting maternal death and disability needs assessment toolkit. Available at: https://www.mailman.columbia.edu/research/averting-maternal-death-and-disability-amdd/toolkit. Accessed: 2015-04-18

*SPA* MEASURE DHS Service Provision Assessment (SPA). Updated June 2012. Available at: http://www.measuredhs.com/What-We-Do/Survey-Types/SPA.cfm. Accessed: 2015-04-10

*SARA* World Health Organization Service availability and readiness assessment. Version 2.2 December 2014. Available at: http://www.who.int/healthinfo/systems/sara_reference_manual/en/Accessed: 2015-04-18

*FASQ* MEASURE Evaluation’s Facility Audit of Service Quality. Version 1. 25 Nov 2008. Available at MEASURE Evaluation IHFAN (International Health Facility Assessment Network): http://ihfan.org. DDI TEMP FASQ 2008 v01. Accessed: 2015-04-26

*HFC* Japan International Cooperation Agency Health Facility Census. Available at MEASURE Evaluation IHFAN (International Health Facility Assessment Network): http://ihfan.org. Zambia 2008 HFC TEMP 2008-v02. Accessed: 2015-04-26

### Application to Zambia

We then used the Zambia HFC to evaluate abortion service availability and coverage using our proposed signal functions. Data to determine PAC and TOP services came from two sets of questions: one on provision of FP and PAC services, asked of all facilities, and the other on EmOC, asked of the subset of facilities that did deliveries. In both cases, staff were asked whether the facility could provide a given service or perform a given function. This method of asking about theoretical capability overestimates actual functioning [17, 35], so adding more specific questions, such as whether a given function has been performed within the last 3 or 6 months, as if often done in EmOC assessments, reduces estimates of capability. We did not have information on actual performance in a recent recall period, but we added criteria on staffing, opening hours, communication tools and referral capability to our classification, partly to make our assessment more stringent. We used a similar approach previously for delivery and antenatal care [23, 36].

Of the 1421 facilities in the HFC dataset, 1369 (96 %) had data on both FP and delivery care provision. Of these, 1130 facilities (one of the 1131 facilities we reported on previously was missing data on provision of family planning) offered delivery care. These facilities were asked their capability to provide the eight EmOC signal functions including manual removal of retained products, parenteral antibiotics, parenteral oxytocics, caesarean section (surgical capability), and blood transfusion among others. The 239 facilities that did not provide delivery care were asked whether they provided PAC, but not about EmOC signal functions. Twenty-two had missing data for this variable.

We used data from the facilities that provided delivery care (and thus had information on both the general PAC question and the specific signal functions) to assess the validity of using the PAC question alone. There were 834 delivery facilities (74 % of 1130) that reported doing PAC, of which 69 % (578) stated they could remove retained products, 81 % (673) could provide parenteral antibiotics, 67 % (557) could provide parenteral oxytocics, and 43 % (360) could do all three functions. Stratified by facility level, 94 % (81) of hospitals that said they provided PAC also said they provided all three functions, compared to 37 % (267) among health centres and 39 % (12) among health posts.

Nevertheless, despite the low validity of the PAC question at lower levels, we assumed that facilities that claimed to provide PAC services actually did so, if we did not have information on signal functions. To be consistent and not disadvantage facilities with more information available, we classified facilities offering all three PAC signal functions as able to provide PAC even if they answered “No” to the PAC question (*n* = 49). Other missing responses for the three signal function variables (<1 % among delivery facilities) were coded as “no”, in other words, as an inability to provide a particular intervention. However, for blood transfusion and surgical capability (measured via caesarean section), which are both required for comprehensive PAC, we assumed facilities with missing information were unable to provide these functions, including for non-delivery facilities where this information was not asked.

The question on 24/7 staffing was also only asked of delivery facilities and was thus missing in 56 non-delivery facilities that reported providing PAC. While such facilities were unlikely to provide 24/7 PAC services, we nevertheless assumed they had such opening times. Where staffing information was entirely missing (in 160 facilities, 12 %), we assumed the required staff members were not available.

To categorize services in Zambia, we allowed for three scenarios:

1. Zambian law scenario (non-emergency)
  All signal functions required, including FP; three doctors in the facility required for legal TOP procedures in non-emergencies. Family planning (as in Table 1) was required because it is best practice for abortion service provision [2, 3]. Three doctors were required because Zambia’s abortion law permits pregnancy termination on a wide range of health and socioeconomic grounds but requires in a non-emergency that abortion be performed with the consent of three registered medical practitioners, one of whom must be a specialist with expertise relating to the case. In emergency situations, consent from only one physician is needed [37], though some providers assume that the risks posed by unsafe abortion mean all cases are emergencies [38]. Because Zambian law makes no specification for PAC [38], this scenario was only applied to TOP.
2. Best practice scenario
  All signal functions required, including FP; three doctors were not needed for TOP, rather one mid-level health professional was adequate, because mid-level providers have been shown effective for this task [39–42]. Family planning was required because this is best practice for abortion service provision.
3. Minimal scenario
  All signal functions except FP required; as in best-practice scenario, only one mid-level health professional (not three doctors) was needed for TOP. This minimal approach focused on the provision of the abortion itself or management of complications, without ensuring an integrated service.

We used a range of analytical measures to report on facility capability, estimate national and ward-level populations, and map facilities in relation to the population distribution. To describe facility capability, we used frequencies and percentages. Missing data were handled as described above.

The decennial 2000 Zambian Census of Population and Housing [43] contains population numbers down to the ward level, with geographic data on administrative boundaries (provinces, districts, constituencies, and wards) and population growth rates by district. We used the district-level population growth rates to compute ward populations in 2005. To compute the total Zambian population in 2005 for Table 4, we used the national rate of population growth of 2.85 % per year between 2000 and 2010, obtained from the decennial 2010 Zambian Census of Population and Housing.

We mapped health facilities and ward areas in the geographic information system platform ArcGIS 9.2 (Esri, Redlands, California, USA) and created circles of 15 km radius around each TOP or PAC facility to calculate the proportion of total area covered and the proportion of the ward population within 15 km of services. Lacking higher resolution data, we had to assume an even spatial population distribution inside wards. In rural Zambia, motorized transport is scarce [44, 45] and around 2005, only 1 % of households owned any [46], which means women often had to walk. We used 15 km distance from services to conform to the UN benchmark of 3 hours of travel time [20], assuming a walking speed of 5 km per hour. Geo-location data (Global Positioning System (GPS) coordinates) were available for 1344 of the 1369 facilities; most of those with missing geographic coordinates were military facilities.

Ethical approval for the secondary data analysis was granted by the London School of Hygiene & Tropical Medicine ethics committee on 03 July 2007 (application number 5172).

## Results

Of the 1369 Zambian health facilities, 65 % reported providing PAC services, 61 % could perform vacuum aspiration and 71 % give parenteral antibiotics. Most hospitals reported they could provide blood transfusion and Caesarean section (87 % and 80 % respectively). Condoms and injectable contraceptives were available in nearly all health centres and posts, but only in around half of the hospitals. Emergency contraceptives were available in 19 % of facilities. Only 28 % of facilities offered FP 7 days per week. Virtually all hospitals and 35 % of health centres had three health professionals registered, but only 42 % of hospitals and virtually none of the health centres and posts had three doctors on their staff list, who would have been all required to sign for a legal TOP, assuming it was not an emergency (Table 3).

**Table 3:** Availability of abortion signal functions by level of facility: Zambia Health Facility Census 2005

	Total^a^	Hospital	Health centre	Health post
	*N* = 1369	*N* = 97	*N* = 1182	*N* = 89
Medication abortion	Not measured			
D&C or D&E	Not measured			
PAC service	65 %	91 %	65 %	39 %
Intravenous fluids	Not measured			
Removal of retained products (Vacuum aspiration)^b^	61 %	98 %	58 %	64 %
Parenteral antibiotics^b^	76 % (63–80 %)	99 % (91–99 %)	75 % (62–79 %)	67 % (38–81 %)
Parenteral oxytocic drugs (Uterotonics)^b^	63 %	97 %	61 %	47 %
Blood transfusion^b^	8 %	87 %	2 %	0 %
Caesarean section (Surgical/laparotomy capability)^b^	7 %	80 %	1 %	0 %
Emergency contraceptives	19 %	26 %	19 %	15 %
Male condoms	93 %	57 %	96 %	92 %
Female condoms	27 %	26 %	27 %	27 %
Spermicides	2 %	6 %	2 %	1 %
Progesterone only pills	76 %	45 %	79 %	73 %
Injectables	86 %	47 %	90 %	80 %
Oral contraceptive pills	91 %	51 %	95 %	89 %
IUD	6 %	31 %	4 %	2 %
Implants	3 %	20 %	2 %	1 %
Female sterilization	5 %	60 %	1 %	0
Male sterilization	3 %	31 %	1 %	0
Family planning available 1+ days per week	95 %	71 %	97 %	91 %
Family planning available 7 days per week	28 %	19 %	29 %	30 %
1+ health professionals (nurses, midwives, clinical officers, or doctors) on duty	85 %	97 %	84 %	79 %
3+ health professionals registered	38 %	98 %	35 %	9 %
3+ doctors registered	4 %	42 %	1 %	0
1+ doctors on duty	9 %	84 %	3 %	0
Health professional with midwifery skills present or on call 24/7^b^	89 % (74–91 %)	96 % (92–96 %)	88 % (74–90 %)	88 % (51–93 %)
Means of communication	61 %	94 %	61 %	27 %
Vehicle for referral^c^	47 %	96 %	44 %	29 %

^a^One facility had an unclassified level

^b^ *N* for these rows is 1130 delivery facilities (90 hospitals, 989 health centres, 50 health posts and 1 unclassified facility), because these questions were not asked for the 239 facilities that did not offer delivery care. For generic functions not unique to delivery care, such as parenteral antibiotics, we could not rule out that facilities for which this question was skipped because they did not do deliveries could not actually provide the function. For this reason we also present a range of percentages (with the column N as denominator) classifying the 239 facilities without information either as all providing the function or as none providing the function for those functions that were likely to be provided in non-delivery facilities

^c^Facility has a vehicle or refers with a vehicle (latter question only asked of the 1130 facilities that did deliveries)

**Fig. 1:**
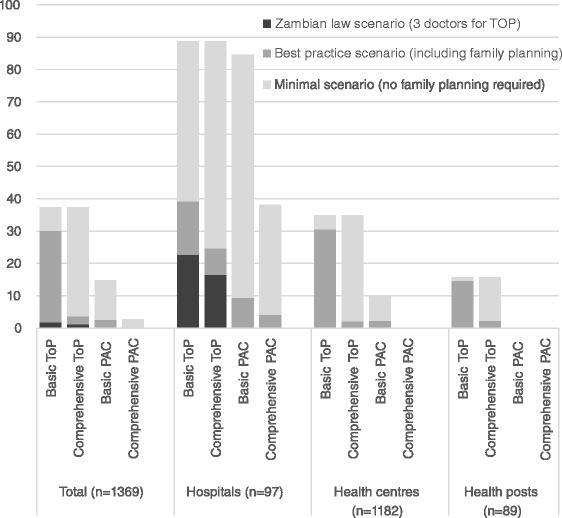
Zambian health facilities’ potential to provide abortion services, percentage by facility level: three scenarios. Percentage of Zambian health facilities potentially providing basic and comprehensive termination of pregnancy (TOP) and post-abortion care (PAC) services, in total and separately for hospitals, health centres and health posts. We consider three scenarios: The minimal scenario just requires abortion signal functions but not family planning signal functions, the best practice scenario requires all (family planning and abortion) signal functions as in Table 1, and the Zambian law scenario (non-emergency) requires three doctors for TOP in addition to all signal functions

Figure 1 presents the percentage of facilities (in total and by level) fulfilling the TOP and PAC criteria under our three scenarios. Under the strictest interpretation, the Zambian law scenario in a non-emergency, requiring three doctors for TOP, only 24 of 1369 facilities (1.8 %) were capable of fulfilling the basic TOP criteria and 16 (1.2 %) of fulfilling the comprehensive TOP criteria. Under the best practice scenario, allowing mid-level providers to perform TOP, these percentages increased to 30.1 % for basic TOP and 3.7 % for comprehensive TOP. Under the minimal scenario, dropping the FP planning requirement, 37.4 % of Zambian facilities fulfilled the criteria for basic and comprehensive TOP. The numbers for basic and comprehensive TOP were the same under this scenario, because the FP requirement was the only difference between these since there were no data on D&E or medical induction in the Zambia HFC. Only 36 facilities (2.6 %) fulfilled basic PAC criteria and 4 facilities (0.3 %) fulfilled comprehensive PAC criteria under the best practice scenario. Under the minimal scenario, where the FP requirement was dropped, this increased to 14.8 % of Zambian facilities fulfilling the criteria for basic PAC and 2.7 % for comprehensive PAC. Availability of services in hospitals was higher than in health centres and health posts, but by best-practice criteria, fewer than 40 % of hospitals would be able to offer basic TOP and fewer than 10 % basic PAC (Fig. 1).

**Fig. 2:**
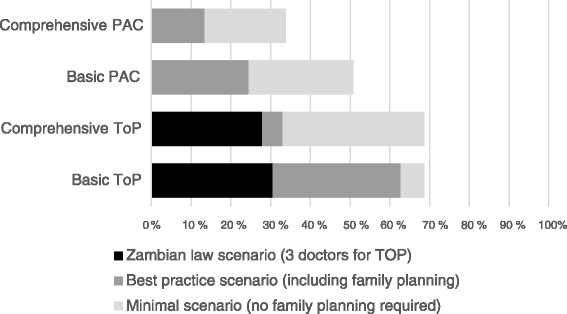
Percentage of Zambian population with access (15 km) to potential abortion services. Percentage of Zambian population living within 15 km of potential basic and comprehensive termination of pregnancy (TOP) and post-abortion care (PAC) services. We consider three scenarios: The minimal scenario just requires abortion signal functions but not family planning signal functions, the best practice scenario requires all signal functions as in Table 1, and the Zambian law scenario (non-emergency) requires three doctors for TOP in addition to all signal functions

Figure 2 shows the proportion of the Zambian population that could have access to TOP and PAC within 15 km, assuming facilities with the right environment, staff, commodities and equipment actually provided the services. Nationally, 86 % of the population lived within 15 km of a health facility, 84 % within 15 km of a facility providing family planning, but only 30 % within 15 km of a facility also employing three doctors. We found that with respect to TOP services, 31 and 28 % of the Zambian population were within 15 km of a facility capable of offering basic or comprehensive TOP, under the Zambian non-emergency law scenario requiring three doctors available in the facility. Under the best practice scenario, the coverage was 63 % of the population within 15 km of basic TOP and 33 % within 15 km of comprehensive TOP. Under the minimal scenario, i.e. if provision of family planning services was not required, then 69 % of the population were within 15 km of both basic and comprehensive TOP services. Coverage within 15 km of PAC services with family planning (best practice scenario) was 25 % for basic PAC and 14 % for comprehensive PAC. If the family planning requirement was dropped (minimal scenario), 51 and 34 % of the population had access to basic and comprehensive PAC respectively.

**Fig. 3:**
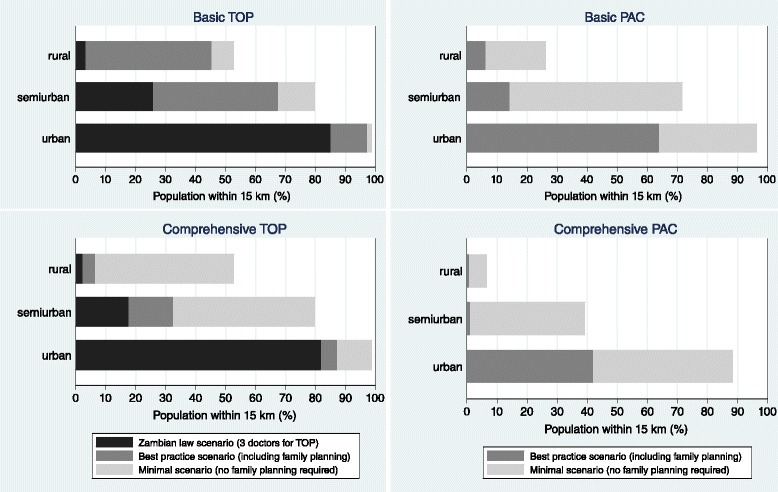
Percentage of Zambian population with access (15 km) to potential abortion services by residence. Percentage of Zambian population living within 15 km of potential basic and comprehensive termination of pregnancy (TOP) and post-abortion care (PAC) services, by place of residence. About 31 % of the population lived in urban, 6 % in semi-urban and 63 % rural wards in 2005. We consider three scenarios: The minimal scenario just requires abortion signal functions but not family planning signal functions, the best practice scenario requires all signal functions as in Table 1, and the Zambian law scenario (non-emergency) requires three doctors for TOP in addition to all signal functions

There were substantial disparities in access to abortion services by place of residence, with only 6 % of the rural population living within 15 km of a facility providing basic PAC, whereas this figure was 64 % for the urban population. While 97 % of the urban population lived within reach of basic TOP services, only 45 % of the rural population did. Requiring three doctors for TOP (Zambian non-emergency law scenario) increased the urban-rural disparity to 85 % urban versus 3 % rural coverage. For comprehensive services, disparities were even larger (Fig. 3).

Table 4 shows SAC services in Zambia as a percentage of required SAC services according to the SAC benchmark [8]. This is low overall (45 %), but is higher for comprehensive service availability (83 %).

**Table 4:** Benchmarks according to Safe Abortion Care (SAC) criteria proposed by Healy and colleagues [8]

Benchmark	Facilities needed given population of Zambia in 2005 was 11,377,000 [43]	Numbers of SAC facilities available^a^	Percent available of SAC needed
1+ comprehensive facilities per 500,000 population	23	19	83 %
4 basic facilities per 500,000 population	91	32	34 %
5 total facilities per 500,000 population	114	51	45 %

^a^According to Table 1 criteria

## Discussion

We modified a set of abortion care functions previously proposed by Healy and colleagues [8], and determined whether existing data collection tools could be used to elicit them. We then applied the signal functions, to the extent possible with secondary data, to Zambia, to assess the potential TOP and PAC capability of health facilities under three different scenarios. Finally, we linked facilities to population census data with GIS to determine the proportion of the Zambian population within reach of TOP and PAC services.

The signal functions approach, first developed by Averting Maternal Death and Disability (AMDD) for EmOC, and elaborated by Healy and colleagues for abortion care, is a useful innovation. Our modification largely emulated Healy and colleagues in the choice of signal functions and in the division of services into basic and comprehensive, but differed in several ways. First, we distinguished between the ability to provide TOP and to provide PAC services, rather than allowing the legal context to determine the extent to which services are “Safe Abortion Care”, as suggested by Healy and colleagues. In settings where induced abortions are restricted by law, Healy et al.’s approach would classify a facility as having basic or comprehensive SAC as long as it provided basic or comprehensive PAC, even if most women resorted to unsafe termination of pregnancy, and the requirements to achieve SAC would thus differ by country, rather than by capability as in our study. We believe the facility capability (and items and staff required) for safe abortion care should be judged on the same standard, irrespective of the legal context, making it more useful for monitoring over time; thus countries providing PAC but not TOP services would appear to do just that, rather than being classified the same as countries providing both TOP and PAC. Second, we defined some signal functions differently. In particular, we elaborated the family planning component more precisely, by specifying a range of methods that needed to be provided at different levels. We added more stringent criteria on family planning because we believe women should have a choice of contraceptive method, in accordance with various quality-of-care frameworks and guidelines [47, 48]. Requiring long-acting methods to be provided as part of comprehensive PAC and TOP will lower capability estimates with such services. By contrast, we relaxed the condition of 24/7 service provision that Healy and colleagues required for all SAC facilities, and applied them only to PAC, but not to TOP services because we judged TOP to be less time-sensitive; we added the requirement of referral capability in basic PAC so that the facility is capable of referring to a facility providing comprehensive PAC in case of severe complications.

### Use of existing tools

We are confident our approach can be adopted widely using existing tools for routinely collected data, with some modifications, as shown in Table 2, and discussed below. A pragmatic adoption of existing tools rather than bespoke tools would still yield longitudinal and internationally comparable measurement and allow for more and better comparative analyses between countries. Multi-purpose facility assessments that collect information on multiple services (e.g. maternal and newborn care, antenatal care, reproductive health and HIV) at the same time are more cost-effective than separate assessments. Among the eight existing multi-country, large-scale facility assessment tools reviewed, five collect information on FP and on removal of retained products as part of EmOC. Having an agreed list of TOP and PAC functions would make it easier to add the relevant questions to existing tools or to modify them to be maximally useful in monitoring progress. The main areas to remedy are to:

- Add questions explicitly asking whether facilities perform TOP or PAC and then include questions on abortion care for these. Currently, all surveys only ask questions related to abortion services if facilities provide services for deliveries (AMDD or HFC); deliveries or newborn care (SPA); normal delivery or BEmOC or CEmOC or newborn care services (SARA) or maternal health services (FASC).
- Add specific questions about the availability of misoprostol and mifepristone, and about the ability to perform D&E and second trimester medical induction. Also ensure FP methods (pills, condoms, injectables, implants, & IUDs) are not all merged in one response (AMDD), so it is possible to distinguish long-acting reversible contraceptives (LARCs). Questions on vacuum aspiration already exist.
- Review wording of functions related to EmOC or surgical services to ensure they also apply to abortions and not just to deliveries. For example, recent tool adaptations have created drawbacks, for example, the SARA and SPA modified the wording to say “remove retained products *after delivery*” (emphasis added), and not after pregnancy ends, thus excluding pregnancy terminations or incomplete abortions, although subsequently SARA reverted back.
- Improve questions on days of service provision, particularly FP provision, which are currently omitted from two surveys (AMDD, SARA). Normally FP does not need 24/7 provision, but in association with PAC, it does, since emergency admissions may not be scheduled. Another survey (SARA) only asked hours, not days, a facility was open.
- Amend questions on staffing in (SPA, SARA) to ensure it is possible to determine whether a medical provider is on duty or on call, since for PAC, these skills are needed 24/7.

### Zambia findings

Using the data from Zambia helped us understand some important aspects of abortion service provision in the country, and particularly how interpretation of a legal requirement greatly impacts on provision of care.

Specifically, we see very clearly that Zambian health services could potentially reach many more women in need of TOP services than women in need of PAC services, particularly if the requirement for three doctors were relaxed by the government, or if all women requesting TOP were deemed emergencies. Greater potential capability to provide TOP was mostly because PAC services required 24/7 facilities, greater numbers of providers to staff such services, and higher provider cadres (doctors) to manage complications; these features were not needed for TOP. Service provision could also be improved by enhancing family planning provision where we found major gaps. In large part, lower-level facilities provided FP, but performed less well on TOP capability. Conversely, hospitals provided TOP and PAC, but failed to a large extent in their capability to also provide FP methods. The desire to have most contraceptive provision near women at the lowest-level facilities, and to avoid expending hospital time providing FP to most women, is understandable. Nevertheless, all TOP and PAC services need to integrate FP, in line with international recommendations, to ensure women using abortion services can prevent subsequent unwanted pregnancy. Low provision of FP by hospitals in Zambia must be remedied [2, 3]. We note our findings are a best case scenario, since we only measured availability of FP and not counselling or provision at the TOP/PAC location.

We further found that vacuum aspiration, a low-cost simple procedure that is important for women’s health and in particular for maternal mortality reduction is only provided in 61 % of health facilities. This is too low, as are the levels of emergency contraception (19 %). Emergency contraceptive provision was introduced decades ago, and increasing access and use is relevant in many programmes, including those aiming to address sexual and gender-based violence.

We also illustrated that when coupled with information on geographic population distribution from a population census, facility assessments with geographic coordinates could be used to calculate population service coverage. This could also be useful for service planning and to identify facilities for upgrading [26] and for assessing inequities. Our coverage figures in Zambia using 15 km straight-line distance were only rough, since we assumed an even population distribution within wards because higher resolution data were not available. The latter will have underestimated access, while assuming 15 km distance was accessible by foot was probably optimistic [49]. Despite these limitations, we could clearly show large inequalities in terms of access by place of residence, exacerbated by adding the criterion of a requirement for three doctors. The urban advantage is in reality probably even larger, considering that smaller private facilities potentially providing abortion care in urban settings might have been missed by the HFC.

The main limitations with respect to the Zambia analysis stem from the problems of using secondary data. The Zambia health facility data are old, and any picture gathered is retrospective, and is mainly useful for tracking improvements over time. We were also forced to make assumptions because of missing data, some of which stemmed from skip patterns where questions were only asked of facilities with deliveries, and because some of what we wanted was not measured or was measured sub-optimally. This may have led us to overestimate capability (for example, we assumed non-delivery facilities reporting provision of PAC actually did provide it, without checking signal functions and we assumed staff present were willing to perform abortions, whereas in practice, some may have ethical objections). At other times we may have underestimated capability. In particular we have some concerns that the strong assumption that missing staff information meant ‘no staff’ was probably unfair on some facilities that would have been classified as PAC or TOP capable otherwise.

### Recommendations for benchmarks

We applied the coverage benchmark proposed by Healy and colleagues to Zambia. This benchmark of five EmOC facilities per 500,000 population is directly transferred from EmOC benchmarks, which in turn assume 15 % of pregnant women will experience complications, of which a subset are abortion complications. We do not see good reasons why the criteria for the delivery care and abortion services should be the same. Best estimates from Zambia suggest there are 39 induced abortions per 1000 women of reproductive age and 20 induced abortions per 100 births [37], so the demand appears high, and the benchmark potentially too low. Risk associated with induced abortion runs along a continuum, and is lowest if evidence-based methods (such as mifepristone/misoprostol) are used in a health facility early in pregnancy and highest if dangerous methods (such as sticks into the uterus or caustic substances taken orally or vaginally) are used late in pregnancy. If all induced abortions used the most dangerous methods, managing the complications would require well above the capability needed for EmOC [50]. Moreover, it seems reasonable to anticipate less PAC will be required as access to safe TOP services increases, since the need for TOP and PAC should go in opposite directions. Better benchmarks would require more data on staff time needed for PAC and TOP and/or bed-days for TOP. It would also be preferable to use the abortion rate rather than the delivery rate to benchmark. We suggest benchmarks are an area for future research, although it may be difficult given that abortion estimates are often imprecise.

Applying the SAC benchmark of five facilities per 500,000 population indicates that 45 % of need is met in Zambia, but for the reasons above, this indicator is difficult to interpret. Using distance to assess coverage of TOP and PAC is a possible solution (Fig. 2) and suggests coverage is poorer than 45 %. We would also recommend creating separate estimates for rural and urban areas, as we have done. Obtaining such coverage estimates in other settings requires similar health facility censuses with geographic coordinates rather than samples of facilities.

Our approach can also be used to model the impact of policy and legal changes. For example, with these data, Zambian policy makers can see the potential impact on equity and coverage of changes in the law of requiring three doctors for TOP in non-emergencies. Such data could also be used to show changes in the proportion of areas (such as urban/rural or districts/regions) that are able to provide TOP and PAC services at basic and comprehensive levels over time, as well as changes in the proportion of women with access to such services.

## Conclusion

In conclusion, several ongoing multi-country efforts such as SPA, SARA and AMDD-UNFPA facility assessments, already produce data that with some modifications can be used to produce analyses similar to ours. These analyses are useful for describing available services. The recommendations in this article aim to contribute to adapting a set of signal functions for abortion care, and for both emergency PAC and routine TOP care. These can be measured with existing tools at different levels of sophistication, to serve a range of purposes and can eventually contribute to improving the quality of reproductive health services in low- and middle-income countries, helping to achieve integration and universal coverage with reproductive and sexual health services. This is timely, given renewed calls to increase access to such services [51, 52].

## Abbreviations

AMDD: averting maternal death and disability
D&C: dilation and curettage
D&E: dilation and evacuation
DFID: Department for International Development
EmOC: emergency obstetric care
FASQ: facility audit of service quality
FP: family planning
GPS: global positioning system
HFC: Health Facility Census
IHFAN: International Health Facility Assessment Network
JICA: Japan International Cooperation Agency
LARCs: long-acting reversible contraceptives
PAC: post-abortion care
SAC: safe abortion care
SARA: service availability and readiness assessment
SPA: service provision assessment
TOP: termination of pregnancy
WHO: World Health Organization
UN: United Nations
